# Antimicrobial Stewardship in Surgery: A Literature Bibliometric Analysis

**DOI:** 10.3389/fpubh.2022.847420

**Published:** 2022-04-07

**Authors:** Yang-Xi Liu, Ya Yang, Ke-Jia Le, Zai-Li Zhang, Min Cui, Han Zhong, Zhi-Chun Gu

**Affiliations:** ^1^Department of Pharmacy, Ren Ji Hospital, Shanghai Jiao Tong University School of Medicine, Shanghai, China; ^2^Department of Infection Control, Ren Ji Hospital, Shanghai Jiao Tong University School of Medicine, Shanghai, China

**Keywords:** surgery, bibliometric analysis, hospital management, trend, antimicrobial stewardship (AMS)

## Abstract

**Background:**

Antimicrobial resistance and the dwindling antibiotic development pipeline have resulted in a looming post-antibiotic era. Research related to antimicrobial stewardship (AMS) has grown rapidly in the past decade, especially in the field of surgery. We conducted a bibliometric analysis of these publications. In addition, we aimed to identify research hotspots and infer future research trends.

**Methods:**

We screened global publications on AMS in the surgical field over ten years (between 2011 and 2020) from the Web of Science core collection database. The keywords “antimicrobial or antibiotic”, “stewardship”, “management”, “management strategies”, “programme”, “surgery” and “surgical” were used to search for related papers. VOS viewer, R software, and other machine learning and visualization tools were used to conduct the bibliometric analysis of the publications.

**Results:**

We identified 674 publications on AMS in surgical fields; “antimicrobial stewardship” (with total link strength of 1,096) was the most frequent keyword, and had strong links to “antimicrobial resistance” and “guidelines”. The top 100 most cited papers had a mean citation count of 47.21 (range: 17–1155) citations, which were cited by survey research studies, clinical trials, and observational studies. The highest-ranking and most cited journal was *Clinical Infectious Diseases* with eight publications. Jason G. Newland from Washington University wrote seven papers and was cited 1,282 times. The University of Washington published 17 papers and was cited 1,258 times, with the largest number of publications by author and organization. The USA published 198 papers and cooperated with 21 countries, mainly partnering with Italy, the UK, and Canada. Published articles mainly focused on the current clinical situation regarding surgical AMS management, antibiotic prescription, and antibiotic resistance.

**Conclusions:**

Publications on surgical AMS management have increased in recent decades, with the USA being the most prolific. Epidemiological investigations of surgical-related infections, antibiotic prescriptions, and antibiotic resistance are fast-developing research trends. However, further improvements are still needed according to the recommendations gained from the bibliometric analysis.

## Introduction

Antibiotics have made significant contributions to the treatment of infectious diseases. For example, sulfonamides, penicillin, and streptomycin became available in the 1930s, after which mortality due to pneumonia (then the leading cause of death) declined by approximately 30% (1). However, antimicrobial resistance due to the extensive use of antibiotics in recent decades has resulted in increased morbidity, mortality, and economic burden, and is a significant global threat. Antibiotic overuse is a growing concern, and a multifaceted approach to prevent, control, and decrease antimicrobial resistance is urgently needed.

Antimicrobial stewardship (AMS), defined as “coordinated interventions designed to improve and measure the appropriate use of antibiotics by promoting the selection of the optimal antibiotic drug regimen, including dosing, duration of therapy, and route of administration”, has been widely accepted in recent decades (2). The purpose of AMS is to achieve the best clinical outcomes related to antibiotics and reduce the excessive costs caused by the irrational use of antimicrobial drugs (3). Controlling infections with antibiotics has an impact on all clinical areas, especially in the field of surgery. Prophylactic antibiotic usage and post-surgical infection treatment both play prominent roles in preventing perioperative infection such as surgical site infection (SSI) (4). As such, AMS in the surgical field is particularly important and the rational use of antibiotics across hospital admissions is viewed as a fiduciary responsibility. The National Health and Family Planning Commission (NHFPC, originally called the Ministry of Health) of China issued a “Notice on further strengthening the administration of clinical application of antibiotics” in 2015, which put forward a detailed appraisal index and formal requirements for clinical application management of antibiotics (5). Since 2016, hospital AMS management has been assessed in accordance with the aforementioned document since 2016 in China.

Bibliometrics is a quantitative tool for analyzing published literature on a specific topic via mathematical methods; it qualitatively and quantitatively describes the details of document features which include the authors of articles, the journals where the works were published, and the number of times they are later cited. It is particularly suitable for scientific publication mapping when research is numerous and fragmented over a period of time (6). The Web of Science (WoS) core database is the world's most convinced publisher-independent global citation database. It also includes the most important research publications and provides analysis tools and primary data for further analysis (7). The use of bibliometrics, based on the WoS core database, is gradually extending to all disciplines.

There have been no publications utilizing bibliometric analysis of AMS in the surgical field to date. As AMS is widely used in surgery, it is important to perform a comprehensive literature analysis to obtain more knowledge on AMS developments in surgical field based on the latest research publications. Additionally, the current status of AMS management in surgery is of major significance to the rational use of antibiotics in the future. Our former studies have finished to build a management and control mode in comprehensive hospital AMS (8), however, AMS management from surgical perspective is lacking. Therefore, we performed this study to provide a structured analysis of AMS research in the surgical field both from macroscopic and microscopic aspects, to infer future trends and identify shifts in this field.

## Methods

### Research Design

We used a bibliometric method to analyze publications regarding AMS in the surgical field. The aim of using the bibliometric method is to formulate a systematic evaluation of various scientific publications to gain rudimentary knowledge of antibiotic management in hospital surgical departments. Furthermore, the analysis of our study focused on various indicators related to article influence, such as publication output and citations. Data was analyzed and visualized with network maps revealing the development of AMS over time in the surgical field.

### Bibliometric Analysis

The bibliometric analysis of our study followed procedural guidelines from a previous publication (9). We focused on searching for articles in the WoS core collection database using comprehensive search strategy to cover more publications. In our study, search terms included “antimicrobial/antibiotic stewardship”, “antimicrobial/antibiotic management”, “antimicrobial/antibiotic management strategies”, “antimicrobial/antibiotic stewardship programme” and “surgery/surgical”, according to previous studies (7, 10, 11). Additionally, language was restricted to English, and document type was restricted to original articles published from January 1, 2011 to December 31, 2020.

### Data Analyses

The analytical tool, VOSviewer (version 1.6.10, Leiden University, Netherlands), was used to analyze the co-authorship, co-occurrence, citation, bibliographic coupling, co-citation, and themes from each publication. For all the analyzed contents except theme and trend, we choose the full counting method, binary counting method were used for theme and trend analysis. In co-authorship analysis, the included articles were with the minimum number of 5 documents and 0 citations. In co-occurrence analysis of keywords, all keywords were included and keywords occurring over 15 times were included into the photograph. In coupling and co-citations analysis, we included documents occurring over 40 times into analysis. While title and abstract were the extracted fields for theme and trend analysis, meanwhile the minimum number of occurrences of a term was 10 and top 50 terms with highest score were selected into cluster.

The online analysis platform of literature metrology (http://webofscience.com/) was utilized to determine the national publication sum and relationship network. R software (version 3.6.1; The R Foundation, Vienna, Austria) was used for further analysis of the literature. Other unspecified cases use the default parameters.

## Results

### Bibliometric Analysis

**Figure 1:**
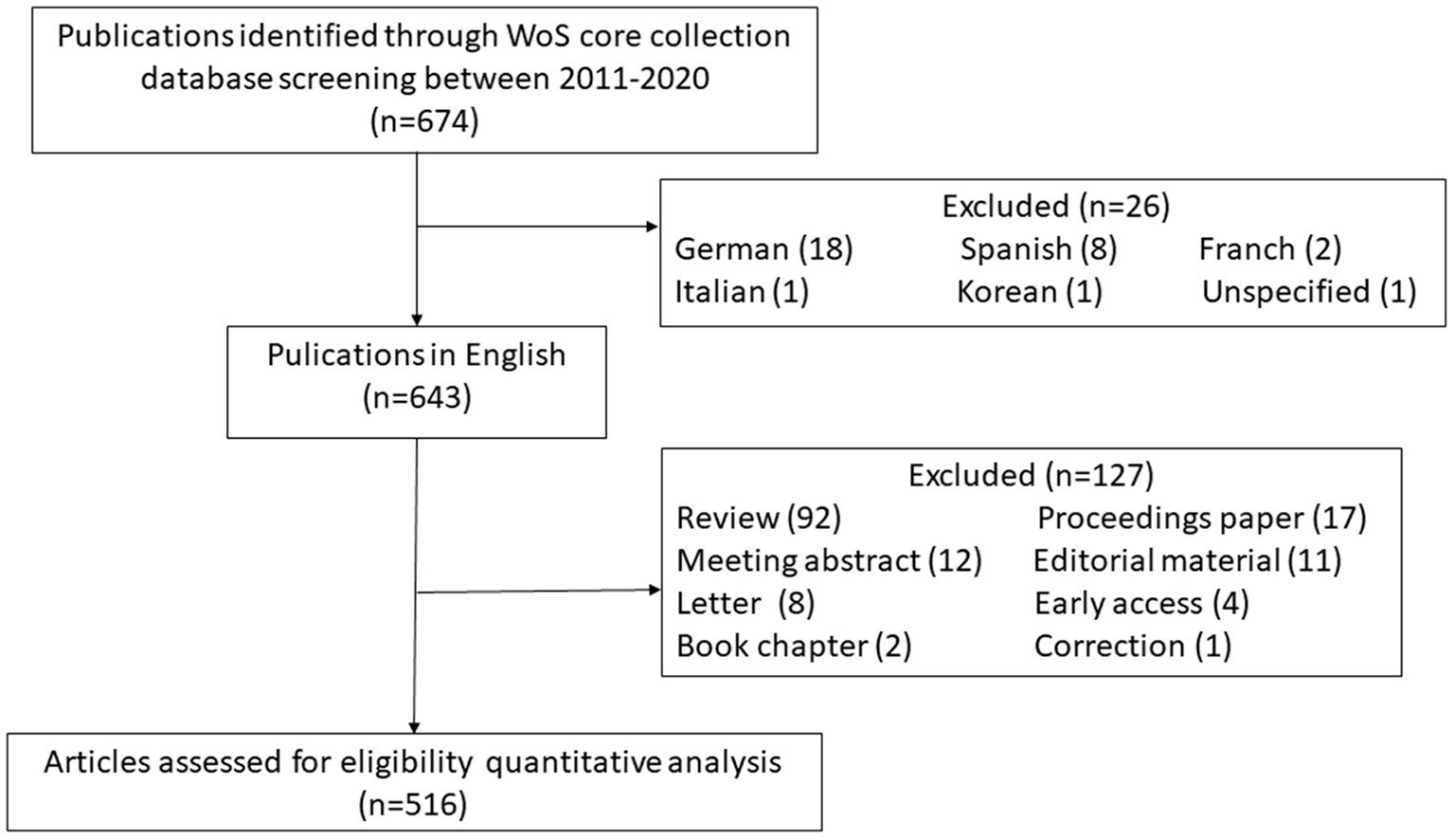
Flow diagram of included publications. WoS, web of science.

Two authors independently completed the retrieval strategy. Full data, including the title, authors, abstract, and keywords, were collected from publications from the WoS core collection database. Figure 1 shows a flowchart illustrated the actions schedule.

#### Overview of Publications

A total of 674 publications on the topic of AMS in surgical departments were identified in the WoS database between 2011 and 2020. These publications included 544 (80.7%) articles, 87 (13.8%) reviews, 17 (2.5%) proceedings papers, 13 (1.9%) editorials, and 27 other forms of publications, including meeting abstracts, letters, and early access manuscripts. Among them, 156 (22.5%) were published in 2020, which encompassed the largest number of publications. A majority of the publications (643, 95.4%) were written in English, followed by 18 (2.7%) publications written in German and 8 (1.2%) written in Spanish. The country with the largest number of publications was the United States (253, 37.5%), followed by the UK (77, 11.4%), and Germany (63, 9.3%). In all published papers, 331 (49.1%) were open access. As show in Figure 1, publications were screened according to the inclusion and exclusion criteria. Finally, a total of 516 articles that were written in English, were included in the keyword analysis.

#### Co-authorship Analysis

**Figure 2:**
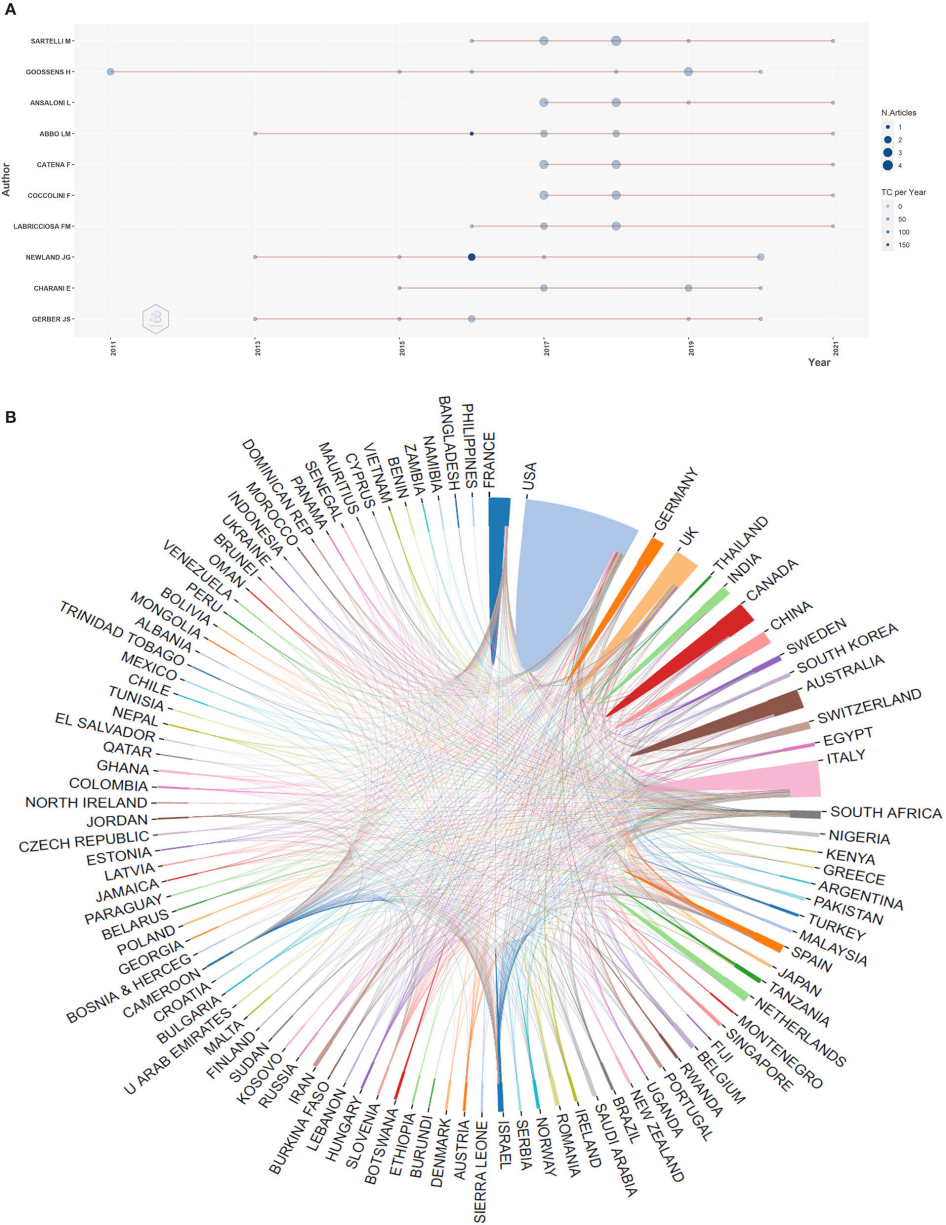
Bibliometric analysis of authors and countries/regions. **(A)** Top 10 authors' productions over time. N. articles, Numbers of articles; TC per year, total citation per year. Among authors, Sartelli M was shortened of Massimo Sartelli, he was the author with the highest number of publications; Goossens H was shortened of Herman Goossens, he was most active author over time; Newland JG was shortened of Jason G. Newland, he was the most cited author. **(B)** Network of countries/regions cooperation. USA was the largest producer.

A total of 3,169 authors participated in the publication of AMS related manuscripts in the surgical field. Among them, Jason G. Newland from Washington University, USA authored seven papers, which mostly focus on the impact of antimicrobial stewardship programs in children's hospitals, and is most highly cited, at 1,282 citations. The total link strength between Newland and other authors was 14. His main collaborators were Jeffrey S. Gerber from the University of Pennsylvania Perelman School of Medicine (in the USA), Adam L. Hersh from the University of Utah (Salt Lake City, USA), and Matthew P. Kronman from Washington University (USA) (Supplementary Figure S1A). We analyzed the manuscripts produced over time for the top 10 prolific authors. Massimo Sartelli from the Department of Surgery, Macerata Hospital, Italy was the author with the highest number of publications; he published four articles in 2018, and mainly focused on the study of surgical infections and sepsis. Herman Goossens from the Laboratory of Medical Microbiology, University of Antwerp, Belgium, had the most articles produced over time focused on antibiotic sensitivity testing. Jason G. Newland was the most cited author even with only two articles (Figure 2A).

According to the domestic and international literature search, it was revealed that 1,217 organizations had published relevant papers; 40 have produced more than five publications. The University of Washington, which published the largest number of related articles, had 17 related papers with 1,258 citations. Their main collaborator was the University of Utah, and most of their research mainly studied the guidelines of AMS in hospitals (link strength of 4.0). Johns Hopkins University and Children's Hospital of Philadelphia were two other important collaborators of the University of Washington (link strength of 3.0, and 3.0, respectively) (Supplementary Figure S1B).

A total of 73 countries have contributed to AMS research. The most productive country was the USA, with 198 publications, in which it collaborated with 21 other countries (the total link strength of the USA was 56). The USA mainly collaborated with Italy, the UK, and Canada (Supplementary Figure S1C). Remarkably, the UK published 64 publications and had the most collaborators (23 countries), with the strongest total link strength of 84. The main partners of the UK were the USA and Italy. Figure 2B displays the cooperative relationship among countries/regions, in which, the USA had a greater interaction with other countries. The top ten most active countries and organizations studying AMS in surgery were listed in Table 1.

**Table 1:** The top 10 most active countries, organizations of AMS in surgery publications.

**Subject**	**Number of publications**	**Count of citations**
**Countries**		
USA	198	3307
UK	64	1032
Italy	38	373
Australia	33	278
Canada	30	612
Germany	29	412
France	26	472
Peoples Republic of China	23	179
India	21	110
Switzerland	17	186
**Organizations**		
University of Washington	17	1258
Johns Hopkins University	13	1360
University of Toronto	11	419
University of Pennsylvania	11	198
Children's Hospital of Philadelphia	11	179
The University of Melbourne	11	123
University of Antwerp	10	269
Harvard Medical School	9	65
Karolinska Institutet	9	58
University of Utah	8	1305
Imperial College London	8	137
University of Colorado	8	106
Monash University	8	51

#### Keywords

**Figure 3:**
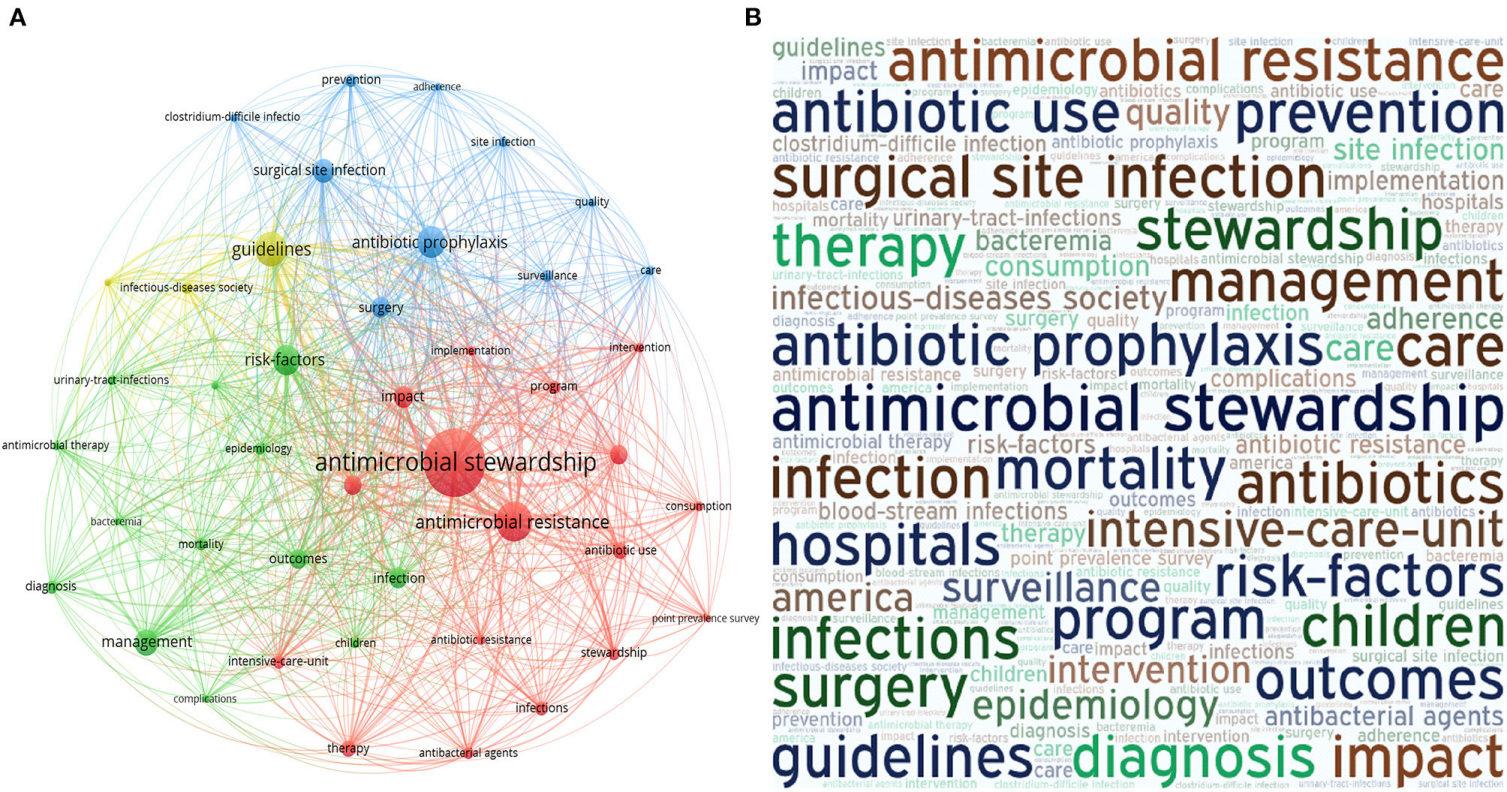
Bibliometric analysis of the keywords in publications of AMS in surgery. **(A)** Co-occurrence of keywords. Various nodes represent the frequency of different keywords occurrences. Curves between the nodes reveal their co-occurrence in the same publication. When the greater the number of co-occurrence in two keywords, the distance between two nodes are shorter. Among all keywords, “antimicrobial stewardship” linked with 718 other keywords (total link strength: 3,137) was the most frequency keywords. **(B)** Word cloud. 49 keywords which occurred more than 15 times were enrolled. Font size indicate the frequency of keywords occurrence. “Antimicrobial stewardship”, “antimicrobial resistance” occurred most frequently.

A total of 516 articles that were written in English, were included in the keyword analysis. Keywords in the articles were provided by the authors and those that occurred more than fifteen times in the WoS core database were enrolled in our analysis. Among 1,854 keywords, 49 keywords met the threshold. The top three most frequently used keywords were “antimicrobial stewardship” (total link strength of 693), “antimicrobial resistance” (total link strength of 410) and “guidelines” (total link strength of 396). The link strength between “antimicrobial stewardship” with “antimicrobial resistance” and “guidelines” was 55 and 56, respectively. After this, “Antibiotic prophylaxis”, “risk-factors”, “management”, “surgical site infection” (SSI) and “impact” were the next most frequently used keywords. The total link strength of all included keywords was greater than 3000 (Figure 3A). Keywords that occurred more than 15 times were then used to create a word cloud (Figure 3B).

#### Citations and Publications

The top 100 most-cited articles written in English about AMS in the surgery field are shown in Supplementary Table S1. Most were clinical studies, including survey research, clinical trials, and observational studies. Other articles included guidelines or expert consensus publications. A majority of research articles have focused on the clinical application of antibiotics, the current situation regarding antimicrobial resistance and epidemiology, and the impact of AMS in surgical management. The studies which successfully implemented AMS and deferred antimicrobial resistance (AMR) were shown in Supplementary Table S2. Meanwhile, guidelines and expert consensus mostly concentrated on ways to improve the rational use of antibiotic prescriptions. The mean citation count of the top 100 most-cited articles was 47.21 (range: 17–1155). Most of the top 100 cited papers were published between 2011 and 2017; 42 of them were written by USA scholars.

**Figure 4:**
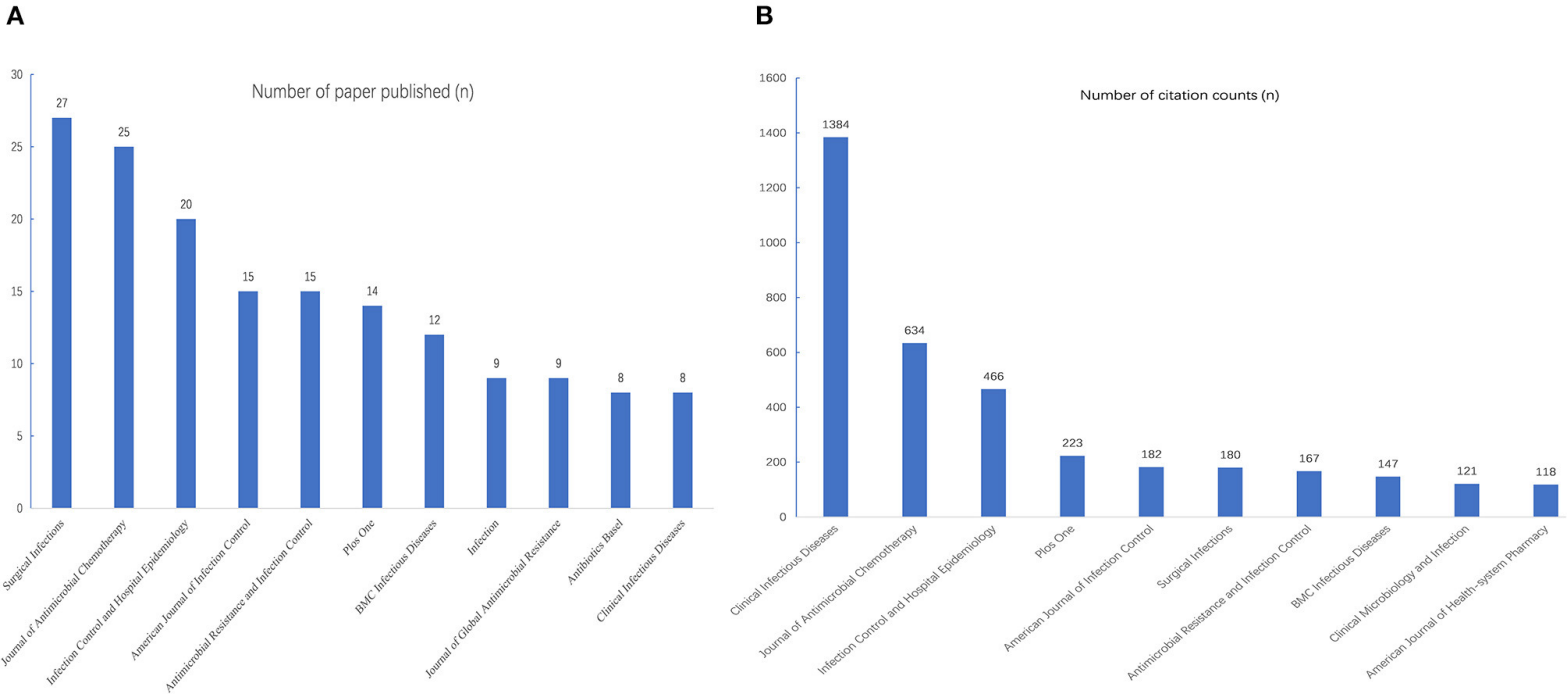
The top ten most active journals. **(A)** The top ten journals with most-cited publications of AMS in surgery; **(B)** The top ten journals with most published papers of AMS in surgery.

**Figure 5:**
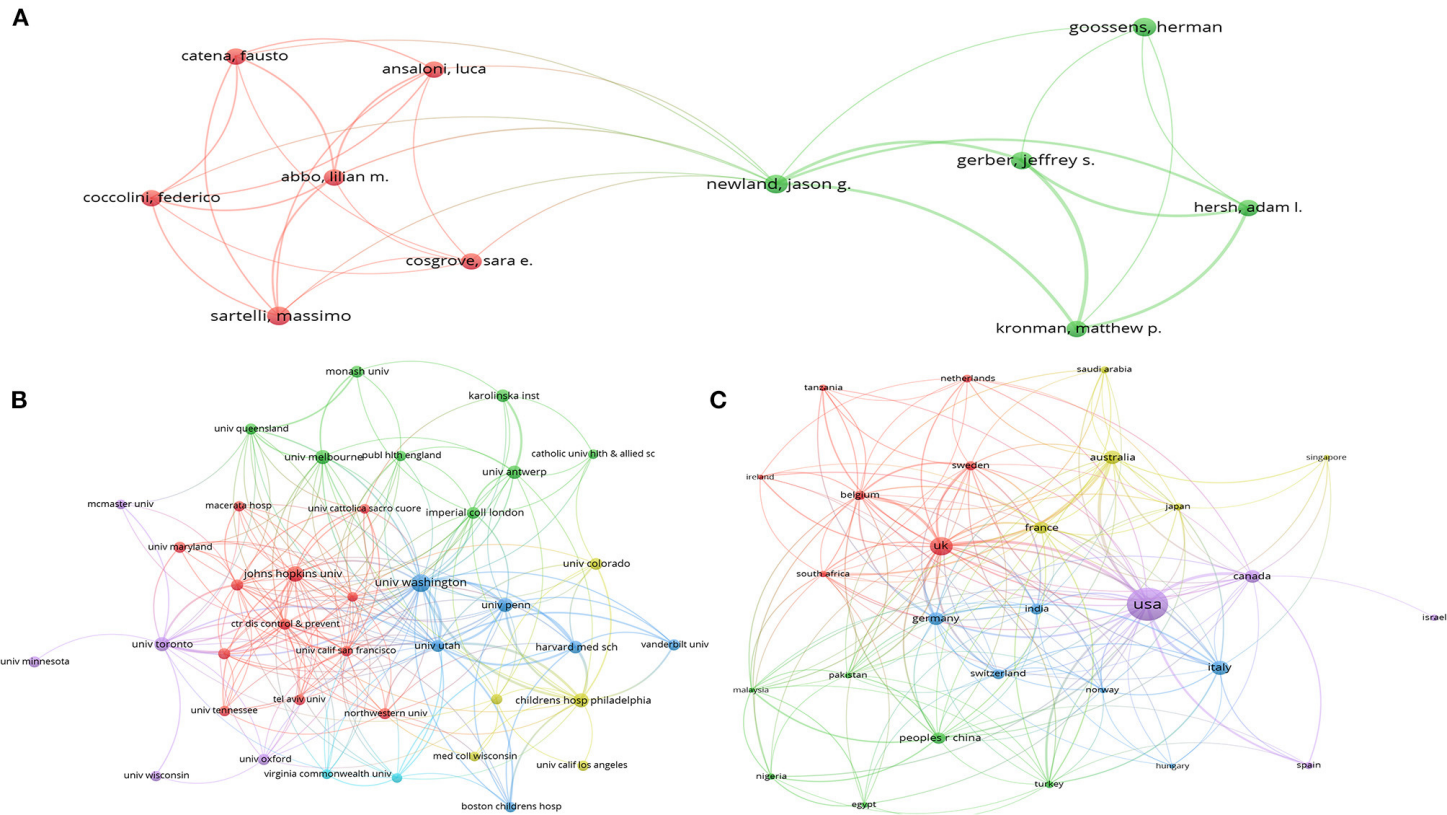
Bibliometric analysis of the citations. **(A)** The citations of authors. Two clusters with different colors were shown. Jason G Newland in green cluster was the most cited author (1,284 times). **(B)** The citations of organizations. J University of Washington in blue cluster (cited 1,258 times) has the largest number of published articles. **(C)** The citations of countries. Five clusters with different colors indicate were shown and USA in purple was cited most (cited 3,307 times). The size of circles indicates the counts of citations.

A total of 190 journals have published papers on AMS in the field of surgery, and 21 of them have published more than five articles. There were 162 articles published in the top ten active journals, accounting for 31.4% of the total published papers in the WoS core database. The highest-ranking journal is *Clinical Infectious Diseases*, which had an impact factor (IF) of 9.079 in 2020, and eight papers were published in this journal during the study period. It is also the most cited journal, with 1,348 citations (Figure 4). The journal *Antibiotics Basel and Clinical Infectious Diseases* shared the first place of the most productive journal with 8 articles. A total of 198 papers from the USA have been cited 3,307 times, and the total link strength of this is 149 (Figure 5).

#### Coupling and Co-citations

**Figure 6:**
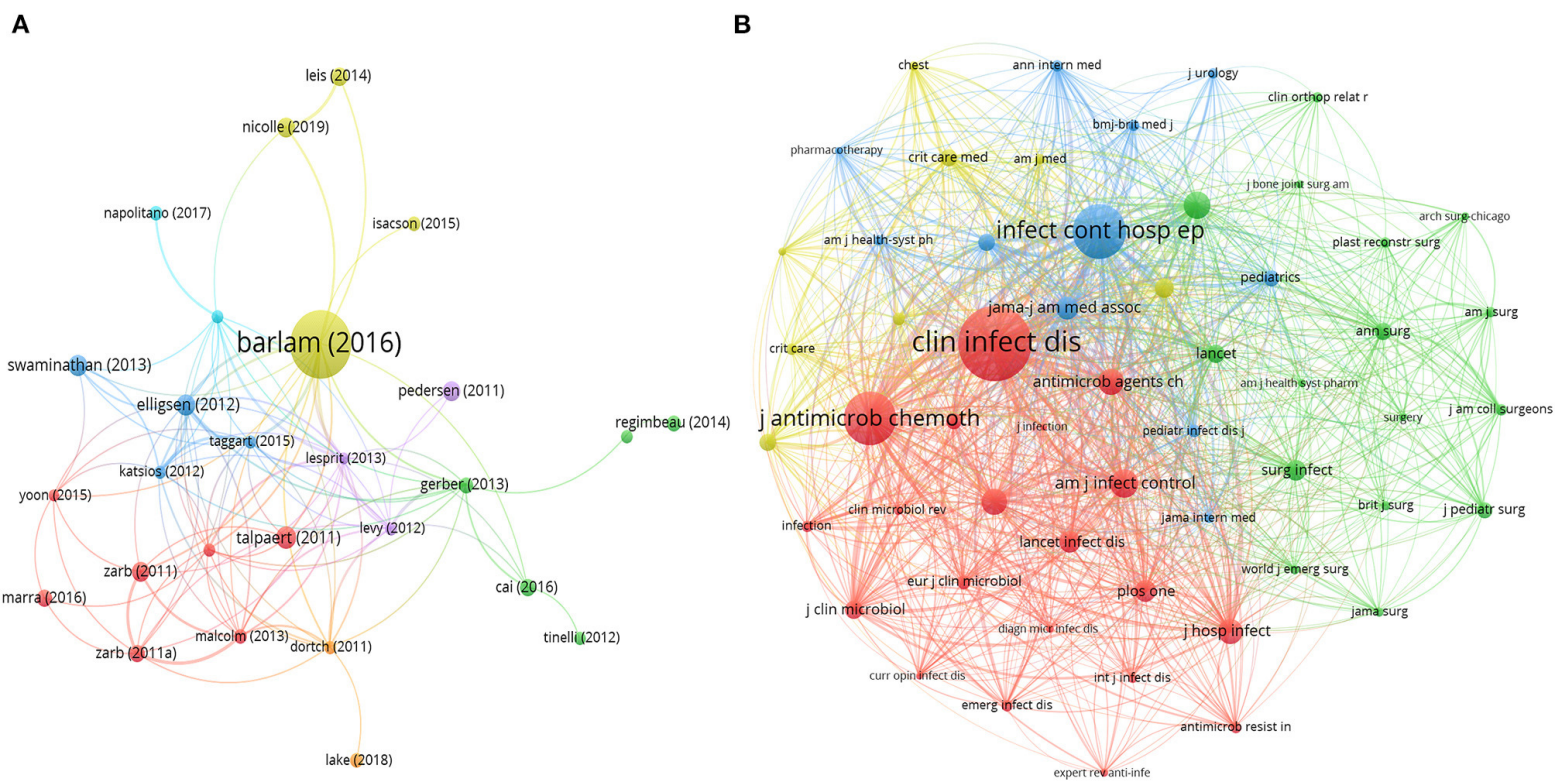
Bibliometric analysis of the bibliographic coupling and co-citation. **(A)** Bibliographic coupling map of documents, 8 cluster with different color represent the different study area and node indicate different articles included. The largest node was barlam (2016) with 1,109 citations and 55 total link strength in cluster 6; **(B)** co-citation map of sources, 4 cluster with different color represent source journals included in different study area. The largest cluster (cluster in red, 20 items) included the largest node (“clin infect dis” with 972 citations and 54 links with other source journals). In both figures, the size of the nodes indicates the counts of co-citations and the distance between two nodes represent their correlation.

The results of the bibliographic coupling analysis are presented in Figure 6A. Seven clusters with 31 documents were obtained from the document analysis. Cluster 4 (in yellow) includes four items, and its research area was the impact of antimicrobial stewardship. The representative paper in Cluster 4, published in *Clinical Infectious Diseases*, provided a guideline for implementing an antimicrobial stewardship program, with 1,109 citations and a total link strength of 46.

Three clusters were obtained from the analysis of sources that were colored in red, green, and blue (Supplementary Figure S2A). Four clusters of co-cited references were obtained through bibliometric analysis (Supplementary Figure S2B). In co-citations analysis, there were four clusters represented the source journals with different research fields: the assessment of AMS policies to reduce SSI and its endemic burden, the relationship between antibiotic prescribing practices with antimicrobial consumption or antibiotic resistance, the current status of antimicrobial use in surgical departments, and the effect of AMS management (Figure 6B).

In our co-cited source analysis, the largest cluster (colored in red) contained 20 items, and the representative journal were the *Clinical Infection Disease* (link strength of 21,597,972 citations) and *Journal of Antimicrobial Chemotherapy* (link strength of 13,580,602 citations). Although Cluster 2 (colored in blue) was contained less items (16 items), the representative journal *Infection Control and Hospital Epidemiology* (link strength of 17,573,632 citations) was highly significant cited.

#### Themes and Trend Topics

**Figure 7:**
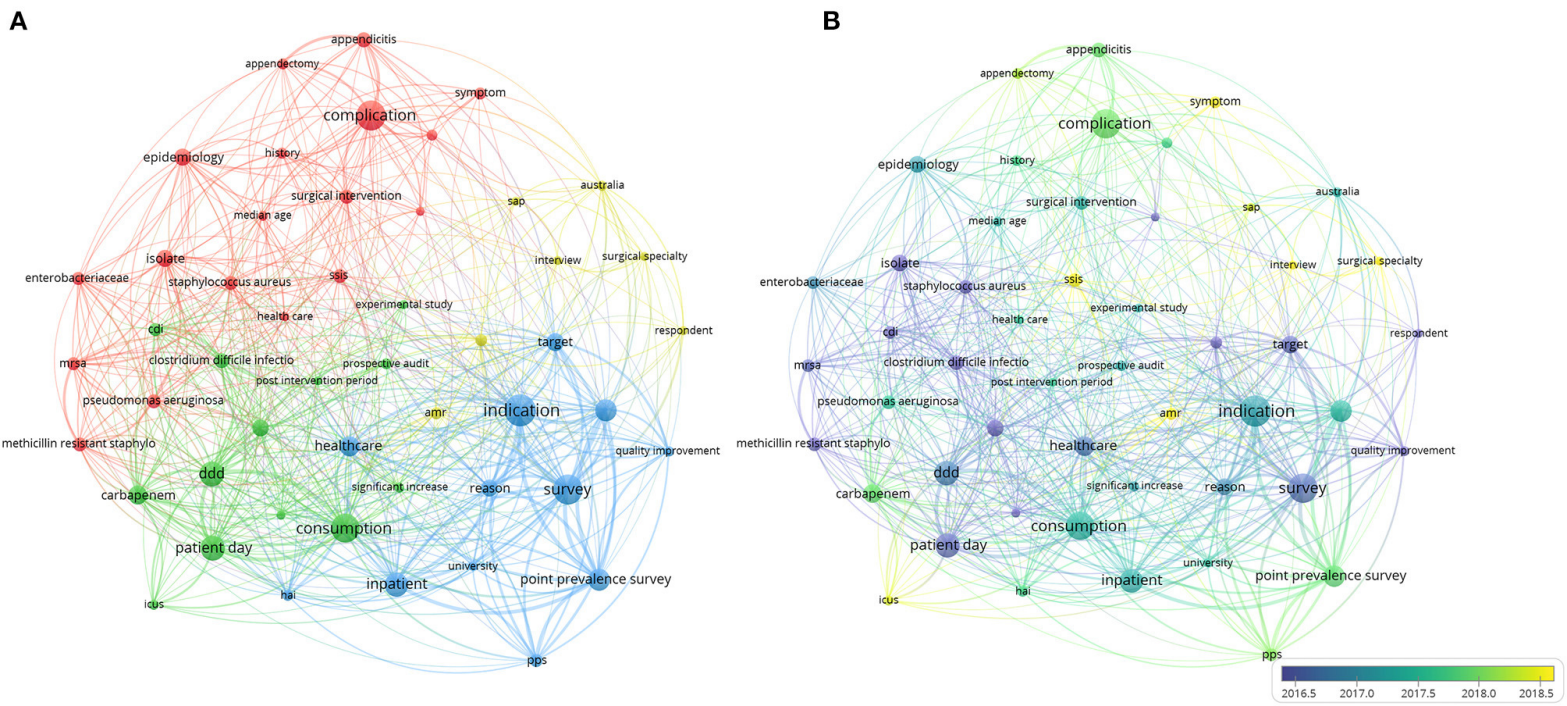
Bibliometric analysis of themes. **(A)** Distribution map of the themes. Four clusters in green, bule, red and yellow were shown in the map, the nodes with same color represented a similar topic according to titles and abstracts. **(B)** Network map of the trend topics. The current publications in different color from purple to yellow. The size of the nodes demonstrates the frequency of appearance as the keywords and the distance between the two circles indicates their correlation.

As shown in Figure 7A, four theme clusters of AMS were found in surgical studies. The green cluster showed the impact of AMS management in clinical practice, including the consumption of antibiotic, carbapenem usage and the result of AMS application such as significant reduction in one particular field. The blue cluster represented where and how AMS management applied. The yellow cluster indicated investigation of AMS management in surgical specialty was the other important themes. While the green cluster mainly focused on the epidemiology of pathogenic microorganism and the complexity in surgical AMS, such as the isolated pathogens isolated and related surgeries. Figure 7B showed the trends of these topics. A variety of color ranging from purple to yellow illustrated different publication times. Recently, studies have mainly focused on reducing SSI, AMR in ICUs and interviews about surgical specialty.

## Discussion

Our study used a bibliometric analysis to draw a comprehensive picture of AMS in surgery research over the past decade. In our current study, 516 articles from the WoS core database were analyzed, and the research output in this area increased every year. The published articles were mostly produced by three countries: the US, the UK, and Italy. The total link strength of AMS with “antimicrobial resistance” and “guidelines” was 11.78 and 13.74, respectively, which demonstrated that the literature mainly focused on strategies to minimize antibiotic resistance. Additionally, the clinical status of infection during surgery and antibiotic usage were also primary study fields. AMS was essentially about optimizing antimicrobial use, however, the expenditure for public health and medical health service system varied from different counties and regions, which implied that implementation strategies for AMS should be modified to adapt local medical environment. We conducted several AMS strategies according to the combination of whole articles reading and AMS experiences of our hospital (Supplementary Figure S3).

### Top-Cited Contributors of AMS in the Surgery Field

AMS can successfully enhance the rational application of antibiotics and has been widely adopted by hospital administrations. However, the current situation of AMS in the surgical field remains perturbing because of some reasons as follows: First, standardized antibiotic recommendation for surgical infection was lack; second, there were limited researches on patient-centered effective AMS interventions; third, most of the studies were single center trial or observational study, therefore evidence of high quality was absent. Our study showed that the affiliated hospital of university (such as the University of Washington and its partner) have contributed extensive research literature to provide useful recommendations for AMS management in surgery. The phenomenon demonstrated that management research and innovation were important work in an affiliated hospital of a comprehensive university Dr. Jason G. Newland, from St. Louis Children's Hospital at Washington University, was the top cited author (with a citation count of 1,282). His investigative research concentrated on the dissemination and implementation of AMS for hospitalized children, which provided sufficient evidence to improve antibiotic use in pediatric surgery (12–14). Meanwhile, guidelines for implementing AMS, such as those written by Barlam et al., had the highest number of citations (1,150 citations), and this advocacy work promoted appropriate antibiotic use in surgery (2). Most research of AMS in surgical field cited this guideline as an authoritative document to complete specific details in hospital management. The majority of the top 10 most-active institutions and authors were located in the USA, where we assumed the influential associations at AMS in surgical field were most located.

### International Collaborations

Organizations in the USA, which produced the most publications of any country, were established as leaders of good practice in the field of AMS surgical management. The USA and the UK maintains a cooperative relationship with 21 and 23 other countries, respectively. Most of their cooperators were from anglosphere countries due to the close association in healthcare. In addition to European countries, such as France and Germany, other developing countries have also played important roles in this field. The countries that study AMS in surgery are concentrated in North America and Europe, while other regions such as Pacific Asia are less involved in studying AMS. This phenomenon might associate with that the level of economic development played important role in healthcare expenditure therefore affect AMS research. Developing countries may not have as much energy as developed countries to invest in surgical AMS. However, the participation of countries at different levels of economic development emphasizes the importance of AMS worldwide and demonstrates that they are taking responsibility to improve the rational use of antibiotics.

### Research Hotspots and Trends

#### Current Clinical Status of Infection in Surgery

Overuse and misuse of antibiotics have been proven to lead to an increase in bacterial resistance (15–17). The incidence of SSIs varies due to the complex interaction of many factors, such as the type of surgery, the number and virulence of contaminating bacteria, and the physical state of patients (18). Therefore, implementing AMS in the surgery department is particularly important to reduce the incidence of resistant pathogens and efficiently improve SSI prevention. (4). The current literature in this field mainly focuses on the epidemiological investigation of pathogens and their antibiotic resistance after surgery. Other studies that have investigated risk factors for SSI and antibiotic prophylaxis management also play an important role in AMS research in the surgical field.

#### Practical Issues in AMS Management

There was not consistent recommendation of antibiotics for different infections related to surgery. This is because the best guides for antibiotic therapy are based on local antibiograms and resistance patterns. However, such information is not readily available, and general principles governing the judicious use of antibiotics should be applied (19). Usually, antibiotic recommendations are based not only on the surgical site and clinical severity of illness, but also on the cost of antibiotics (20). Inappropriate initial administration of antibiotics may result in longer hospital stays, higher mortality rates, higher prevalence of drug-resistant pathogens, and higher cost implications (for example, using an agent to which causative pathogens are not susceptible). Therefore, AMS in different surgery wards is widely accepted, leading to significant reduction in the abuse of antibiotics and patient care costs (1, 2, 4, 18, 21, 22). In our study, we found that the literature focused on the complexity of AMS, because it varied among different diseases and populations. Hence, the topic was scattered into many branches and was primarily concerned with improving the quality of healthcare institutions.

#### The Influence of AMS in Surgery

Prolonged, improper, and unregulated use of antibiotics is a key factor in the rapid rise in resistant pathogens, which further leads to the failure of surgery-related infection treatment (19). Surveillance of antibiotic usage and AMS management in healthcare institutions is crucial to inform and evaluate AMS strategies in surgery. Our results showed that articles were primarily focused on two fields: (1) the effective evaluation of antibiotic use in different inpatient wards. The majority of the articles in this field were concerned about prescription behavior in different wards; (2) the impact of AMS management in clinical practice, researchers were especially interested in how AMS can improve the quality of medical treatment in intensive care units (ICUs) and how to evaluate the economic effect of antibiotics with AMS. The explanation for this phenomenon might be that the ICU and surgical department both have a high incidence of all kinds of infections, and therefore use antibiotics extensively (23–26). It was also demonstrated that these departments were taking action to reduce antibiotic use and limit the emergence of resistance.

### Future Recommendation

An interdisciplinary team targeting to optimizing antibiotic use in surgery was the key determinant of success in implementing AMS. We suggested medical institutions should establish an AMS team including trained administrators, surgeons and nurses, pharmacists, microbiologist, and others if necessary. Moreover, the main procedure of AMS in surgery involved the expert consultation, prior authorization for restricted antibiotics, prospective-audit-with-feedback, and the education in antibiotic use. These AMS procedures required a huge and powerful execution and we emphasized more on interdisciplinary cooperation based on communication. Finally, restricting excess antimicrobial use through AMS team raised a question for the doctors' right to prescribe and we recommended AMS team should balance the reduction excess antibiotic use between without impeding access to antibiotics.

### Strengths and Limitations

Our study is one of the first bibliometric analysis to evaluate publications on AMS in surgery. Results of the bibliometric analysis of citations, co-authors, and other factors illustrated the current research status, hotspots, and future concerns of surgical AMS studies. Nevertheless, this study has several limitations. First, although our study enrolled articles published in English, a small portion of other types of literature, such as guidelines and meta-analyses, were not fully removed. Thus, the results may have a slight inaccuracy due to the impact of repetitive citations. Another significant limitation is the lack of implementation research in this area. Little effort and research funding have been allocated to study how best to achieve large-scale implementation of AMS. Moreover, having few high-quality prospective cohort studies, may limit the quality of the bibliometric analysis.

## Conclusion

Our study is one of the first bibliometric analysis to evaluate publications on AMS in surgery using the WoS core database. These studies emphasized the details of AMS management in surgery, which aims to decrease surgical-related infections and reduce antibiotic resistance. Professionals have identified AMS programs as a key element in the rational use of antibiotics in surgical departments. For now, the management of AMS in surgical gained rapid attention but still needs further refinement.

## Data Availability Statement

The original contributions presented in the study are included in the article/Supplementary Material, further inquiries can be directed to the corresponding author/s.

## Author Contributions

Z-CG and HZ are the guarantors of the entire manuscript. Y-XL and YY contributed to the study conception and design, critical revision of the manuscript for important intellectual content, and final approval of the version to be published. K-JL, Z-LZ, and MC contributed to the data acquisition, analysis, and interpretation. All authors contributed to the article and approved the submitted version.

## Funding

This study was funded by Clinical Science and Technology Innovation Project of Shanghai Shen Kang Hospital Development Center (SHDC12021615), the Clinical Research Innovation and Cultivation Fund of Ren Ji Hospital (RJPY-LX-008), and Research Funds of Shanghai Rising Stars of Medical Talents Youth Development Program-Youth Medical Talents: Clinical Pharmacist Program [SHWSRS (2021) 099], Shanghai Health and Family Planning commission (20194Y0007), and China International Medical Foundation (Z-2018-35-2003).

## Conflict of Interest

The authors declare that the research was conducted in the absence of any commercial or financial relationships that could be construed as a potential conflict of interest.

## Publisher's Note

All claims expressed in this article are solely those of the authors and do not necessarily represent those of their affiliated organizations, or those of the publisher, the editors and the reviewers. Any product that may be evaluated in this article, or claim that may be made by its manufacturer, is not guaranteed or endorsed by the publisher.
