# Supplementary material for: Antimicrobial Stewardship in Surgery: A Literature Bibliometric Analysis
Source: Front Public Health. 2022 Apr 7;10:847420. doi: 10.3389/fpubh.2022.847420 (PMC9021645; doi:10.3389/fpubh.2022.847420)
Supplement: Supplementary file 1 [file Data_Sheet_1.PDF]

Supplementary Table S1. The top 100 most cited articles in the field of surgical AMS

Rank	Title	Journal	Article type	Country of the corresponding author	Publication date	Citations
1	Implementing an Antibiotic Stewardship Program: Guidelines by the Infectious Diseases Society of America and the Society for Healthcare Epidemiology of America	Clinical Infectious Diseases	Guideline	USA	2016/05	1155
2	Impact of guidelines and enhanced antibiotic stewardship on reducing broad-spectrum antibiotic usage and its effect on incidence of Clostridium difficile infection	Journal of Antimicrobial Chemotherapy	Retrospective, quasi-experimental study	UK	2011/09	120
3	Audit and Feedback to Reduce Broad-Spectrum Antibiotic Use among Intensive Care Unit Patients: A Controlled Interrupted Time Series Analysis	Infection Control and Hospital Epidemiology	Prospective, controlled study	Canada	2012/04	113
4	Prevalence and Risk Factors for Acquisition of Carbapenem-Resistant Enterobacteriaceae in the Setting of Endemicity	Infection Control and Hospital Epidemiology	Cohort study with a nested case-control study	USA	2013/08	112
5	ASHP national survey of pharmacy practice in hospital settlings: Prescribing and transcribing-2010	American Journal of Health-system Pharmacy	Questionnaire survey	USA	2011/04	98
6	Identification of targets for quality improvement in antimicrobial prescribing: the web-based ESAC Point Prevalence Survey 2009	Journal of Antimicrobial Chemotherapy	Survey Research	Malta	2011/02	97
7	Reducing Antimicrobial Therapy for Asymptomatic Bacteriuria Among Noncatheterized Inpatients: A Proof-of-Concept Study	Clinical Infectious Diseases	Proof-of-Concept Study	USA	2014/04	89
8	European Surveillance of Antimicrobial Consumption (ESAC) Value of a Point-Prevalence Survey of Antimicrobial Use Across Europe	Drugs	Survey Research	Belgium	2011	75

9	Adherence to European Association of Urology Guidelines on Prophylactic Antibiotics: An Important Step in Antimicrobial Stewardship	European Urology	Before-after study	Italy	2016/02	74
10	Antibiotic prescribing by dentists has increased Why?	Journal of the American Dental Association	Survey Research	UK	2016/05	72
11	Effect of Postoperative Antibiotic Administration on Postoperative Infection Following Cholecystectomy for Acute Calculous Cholecystitis A Randomized Clinical Trial	JAMA- Journal of the American Medical Association	Randomized Clinical trial	France	2014/07	72
12	Identifying Targets for Antimicrobial Stewardship in Children's Hospitals	Infection Control and Hospital Epidemiology	Retrospective Cross-sectional study	USA	2013/12	64
13	Pathogen Distribution and Antimicrobial Resistance Among Pediatric Healthcare-Associated Infections Reported to the National Healthcare Safety Network, 2011-2014	Infection Control and Hospital Epidemiology	Descriptive Study	USA	2018/01	56
14	Outpatient, non-antibiotic management in acute uncomplicated diverticulitis: a prospective study	International Journal of Colorectal Disease	Prospective study	Sweden	2015/09	55
15	Does eosinophilic COPD exacerbation have a better patient outcome than non-eosinophilic in the intensive care unit?	International Journal of Chronic Obstructive Pulmonary Disease	Retrospective observational cohort study	Turkey	2015	53
16	Clostridium difficile disease: Diagnosis, pathogenesis, and treatment update	Surgery	Descriptive Study	USA	2017/08	52
17	From intermittent antibiotic point prevalence surveys to quality improvement: experience in Scottish hospitals	Antimicrobial Resistance and Infection Control	Descriptive Study	Scotland	2013	51
18	Independent predictors of failure of nonoperative management of spinal epidural abscesses	Spine Journal	Retrospective, case-control study	USA	2014/08	50
19	The increasing role of pharmacists in antimicrobial stewardship in English hospitals	Journal of	Questionnaire	UK	2013/11	50

		Antimicrobial Chemotherapy	survey			
20	Microbiology and Antibiotic Management of Orbital Cellulitis	Pediatrics	Retrospective chart study	USA	2011/03	48
21	Antibiotic use in Vietnamese hospitals: A multicenter point-prevalence study	American Journal of Infection Control	Prevalence study	Japan	2012/11	46
22	Infection Reduction Strategies Including Antibiotic Stewardship Protocols in Surgical and Trauma Intensive Care Units Are Associated with Reduced Resistant Gram-Negative Healthcare-Associated Infections	Surgical Infections	Retrospective study	USA	2011/02	46
23	Differential outcome of an antimicrobial stewardship audit and feedback program in two intensive care units: a controlled interrupted time series study	BMC Infectious Diseases	Prospective chort study	Canada	2015/10	44
24	Rates and Appropriateness of Antimicrobial Prescribing at an Academic Children's Hospital, 2007-2010	Infection Control and Hospital Epidemiology	Retrospective study	USA	2012/04	44
25	Implementation of an antimicrobial stewardship program on the medical-surgical service of a 100-bed community hospital	Antimicrobial Resistance and Infection Control	Retrospective study	USA	2012	44
26	Antibiotics-First Versus Surgery for Appendicitis: A US Pilot Randomized Controlled Trial Allowing Outpatient Antibiotic Management	Annals of Emergency Medicine	Multi-center study	USA	2017/07	43
27	Epidemiology and genetic characteristics of extended-spectrum -lactamase-producing Gram-negative bacteria causing urinary tract infections in long-term care facilities	Journal of Antimicrobial Chemotherapy	Prospective case-case control study	Italy	2012/12	43
28	Measuring antimicrobial prescribing quality in Australian hospitals: development and evaluation of a national antimicrobial prescribing survey tool	Journal of Antimicrobial Chemotherapy	Reliability and validity evaluation	Australia	2015/06	42
29	An antimicrobial stewardship program improves antimicrobial treatment by culture site and the quality of antimicrobial prescribing in critically ill patients	Critical Care	Retrospective study	Canada	2012	42

30	Trends of Antibiotic Consumption in Korea According to National Reimbursement Data (2008-2012) A Population-Based Epidemiologic Study	Medicine	Epidemiologic Study	South Korea	2015/11	41
31	Clinical impact of unsolicited post-prescription antibiotic review in surgical and medical wards: a randomized controlled trial	Clinical Microbiology and Infection	Randomized controlled trial	France	2013/02	41
32	Predictors of bla(CTX-M-15) in varieties of Escherichia coli genotypes from humans in community settings in Mwanza, Tanzania	BMC Infectious Diseases	Cross-sectional study	Tanzania	2016/04	40
33	Investigating the cultural and contextual determinants of antimicrobial stewardship programmes across low-, middle- and high-income countries-A qualitative study	Plos One	Qualitative study	UK	2019/01	38
34	Antibiotic Resistance of Commensal Staphylococcus aureus and Coagulase-Negative Staphylococci in an International Cohort of Surgeons: A Prospective Point-Prevalence Study	Plos One	Prospective Study	USA	2016/02	38
35	Behavioral Approach to Appropriate Antimicrobial Prescribing in Hospitals The Dutch Unique Method for Antimicrobial Stewardship (DUMAS) Participatory Intervention Study	JAMA Internal Medicine	Prospective intervention study	Netherlands	2017/08	37
36	What antibiotics for what pathogens? The sensitivity spectrum of isolated strains in an intensive care unit	Science of the Total Environment	Survey Research	France	2019/11	34
37	Significant Reduction of Antibiotic Consumption and Patients' Costs after an Action Plan in China, 2010-2014	Plos One	Survey Research	China	2015/03	34
38	Diabetic foot infections: Current treatment and delaying the post-antibiotic era'	Diabetes-Metabolism Research and Reviews	Practice guideline	UK	2016/01	32
39	Antibiotic management and early discharge from hospital: an economic analysis	Journal of Antimicrobial Chemotherapy	Economic analysis	UK	2012/09	32
40	Strategies for Improving Antimicrobial Use and the Role of Antimicrobial Stewardship Programs	Clinical Infectious Diseases	Practice guideline	USA	2011/08	32
41	Procalcitonin-guided antibiotic therapy: an expert consensus	Clinical Chemistry and Laboratory Medicine	Expert consensus	Italy	2018/08	31

42	Understanding antibiotic decision making in surgery-a qualitative analysis	Clinical Microbiology and Infection	Qualitative study	UK	2017/11	30
43	The Role of Topical Antiseptic Agents Within Antimicrobial Stewardship Strategies for Prevention and Treatment of Surgical Site and Chronic Open Wound Infection	Advances in Wound Care	Expert consensus	UK	2017/02	30
44	Using a simple point-prevalence survey to define appropriate antibiotic prescribing in hospitalised children across the UK	BMJ Open	Cross-sectional study	UK	2016	30
45	There should be no ESKAPE for febrile neutropenic cancer patients: the dearth of effective antibacterial drugs threatens anticancer efficacy	Journal of Antimicrobial Chemotherapy	Research article	Canada	2013/03	30
46	Antibiotic stewardship programmes and the surgeon's role	Journal of Hospital Infection	Expert proposes	Turkey	2015/04	29
47	Perioperative, Postoperative, and Prophylactic Use of Antibiotics in Alloplastic Total Temporomandibular Joint Replacement Surgery: A Survey and Preliminary Guidelines	Journal of Oral and Maxillofacial Surgery	Survey and Guidelines	USA	2011/08	29
48	Antibiotic use in a tertiary healthcare facility in Ghana: a point prevalence survey	Antimicrobial Resistance and Infection Control	Survey research	Ghana	2018/01	28
49	From guidelines to practice: a pharmacist-driven prospective audit and feedback improvement model for peri-operative antibiotic prophylaxis in 34 South African hospitals	Journal of Antimicrobial Chemotherapy	Reliability and validity evaluation	South Africa	2017/04	28
50	Antibiotic usage in German hospitals: results of the second national prevalence study	Journal of Antimicrobial Chemotherapy	Survey research	Germany	2013/12	28
51	Multicenter Study of the Risk Factors for Colonization or Infection with Carbapenem-Resistant Enterobacteriaceae in Children	Antimicrobial Agents and Chemotherapy	Multicenter matched case-control study	USA	2017/12	27

52	A matched-control evaluation of an antifungal bundle in the intensive care unit at a university teaching hospital	International Journal of Clinical Pharmacy	Matched-control evaluation	USA	2013/02	27
53	Variations in Neonatal Antibiotic Use	Pediatrics	Retrospective cohort study	USA	2018/09	26
54	Screening for Beta-Lactam Allergy in Joint Arthroplasty Patients to Improve Surgical Prophylaxis Practice	Journal of Arthroplasty	Cohort study	USA	2017/09	26
55	Antibiotic Use in Small Community Hospitals	Clinical Infectious Diseases	Research article	USA	2016/11	26
56	Antimicrobial Prescribing in Dogs and Cats in Australia: Results of the Australasian Infectious Disease Advisory Panel Survey	Journal of Veterinary Internal Medicine	Survey Research	Australia	2017/07-08	25
57	Staffing for infectious diseases, clinical microbiology and infection control in hospitals in 2015: results of an ESCMID member survey	Clinical Microbiology and Infection	Retrospective study	Israel	2016/09	25
58	Gap Analysis of Infection Control Practices in Low- and Middle-Income Countries	Infection Control and Hospital Epidemiology	Survey Research	USA	2015/10	25
59	Multifaceted intervention to optimize antibiotic use for intra-abdominal infections	Journal of Antimicrobial Chemotherapy	Before-after study	Canada	2015/04	25
60	Practice of switch from intravenous to oral antibiotics	Springerplus	Retrospective observational study	Lebanon	2014/12	25
61	Is switching to an oral antibiotic regimen safe after 2 weeks of intravenous treatment for primary bacterial vertebral osteomyelitis?	BMC Infectious Diseases	Retrospective study	Switzerland	2014/04	25
62	Surgeons Do Not Listen: Evaluation of Compliance with Antimicrobial Stewardship Program Recommendations	American Surgon	Retrospective study	USA	2013/12	24
63	Real-time bacterial fluorescence imaging accurately identifies wounds with moderate-to-heavy bacterial burden	Journal of Wound Care	Cohort study	USA	2019/06	23

64	Healthcare-acquired infections: prevention strategies	Internal Medicine Journal	Research article	Australia	2017/12	23
65	Optimizing preoperative prophylaxis in patients with reported beta-lactam allergy: a novel extension of antimicrobial stewardship	Journal of Antimicrobial Chemotherapy	Research article	Canada	2017/09	23
66	The Global Alliance for Infections in Surgery: defining a model for antimicrobial stewardship-results from an international cross-sectional survey	World Journal of Emergency Surgery	Cross-sectional web-based survey	Italy	2017/08	23
67	Silver-Impregnated Occlusive Dressing Reduces Rates of Acute Periprosthetic Joint Infection After Total Joint Arthroplasty	Journal of Arthroplasty	Retrospective study	USA	2017/03	23
68	Left ventricular assist device exchange for persistent infection: a case series and review of the literature	Transplant Infectious Disease	Review	USA	2014/06	23
69	Antibiotic therapy for pediatric deep neck abscesses: A systematic review	Internal Journal of Pediatric Otorhinolaryngology	Review	USA	2012/11	23
70	The effect of a whole-system approach in an antimicrobial stewardship programme at the Singapore General Hospital	European Journal of Clinical Microbiology & Infectious Diseases	Retrospective study	Singapore	2012/06	23
71	Antimicrobial use in European acute care hospitals: results from the second point prevalence survey (PPS) of healthcare-associated infections and antimicrobial use, 2016 to 2017	Eurosurveillance	Survey research	Sweden	2018/11	22
72	Antibiotic prophylaxis for surgical site infections as a risk factor for infection with Clostridium difficile	Plos One	Retrospective case-control study	USA	2017/06	22
73	Evidence-Based Protocol for Infection Control in Immediate Implant-Based Breast Reconstruction	Annals of Plastic Surgery	Protocol	USA	2012/10	22
74	Trends in Oral Antibiotic Prescription in Dermatology, 2008 to 2016	JAMA Dermatology	Cross-sectional	USA	2019/03	21

			analysis			
75	Incidence of periorbital necrotising fasciitis in the UK population: a BOSU study	British Journal of Ophtalmology	Prospective observational study	UK	2014/09	21
76	Impact of an antimicrobial stewardship program on patients with acute bacterial skin and skin structure infections	American Journal of Health-system Pharmacy	Observational chart review	USA	2014/07	21
77	Antimicrobial management of septic arthritis of the hand and wrist	Infection	Retrospective study	USA	2014/04	21
78	A survey of resident physicians' knowledge regarding urine testing and subsequent antimicrobial treatment	American Journal of Infection Control	Survey Resaerch	USA	2013/10	21
79	Epidemiology of Vancomycin-Resistant Enterococcus faecalis: a Case-Case-Control Study	Antimicrobial Agents and Chemotherapy	Case-Case-Control Study	USA	2013/01	21
80	General treatment principles for fracture-related infection: recommendations from an international expert group	Archives of Orthopaedic and Trauma Surgery	Review	Belgium	2020/08	20
81	A multicenter point prevalence survey of antibiotic use in Punjab, Pakistan: findings and implications	Expert Review of Anti-infective Therapy	Survey Resaerch	USA	2019/04	20
82	Ventilator-associated pneumonia in patients assisted by veno-arterial extracorporeal membrane oxygenation support: Epidemiology and risk factors of treatment failure	Plos One	Retrospective study	France	2018/04	20
83	Extended- Versus Narrower-Spectrum Antibiotics for Appendicitis	Pediatrics	Retrospective cohort study	USA	2016/07	20
84	Choosing Wisely in Healthcare Epidemiology and Antimicrobial Stewardship	Infection Control and Hospital Epidemiology	Practice guidline	USA	2016/07	20
85	Impact of an Antimicrobial Stewardship Program on Patient Safety in Veterans Prescribed Vancomycin	Clinical Therapeutics	Retrospective chart review	USA	2016/03	20

86	Antimicrobial stewardship to optimize the use of antimicrobials for surgical prophylaxis in Egypt: A multicenter pilot intervention study	American Journal of Infection Control	Before-and-after study	Egypt	2015/11	20
87	Antibiotic consumption after implementation of a procalcitonin-guided antimicrobial stewardship programme in surgical patients admitted to an intensive care unit: a retrospective before-and-after analysis	Infection	Before-and-after study	Germany	2015/08	20
88	Laryngectomy Complications Are Associated with Perioperative Antibiotic Choice	Otolarynglogy-head and Neck Surgery	Retrospective analysis	USA	2015/07	20
89	SCIP ping antibiotic prophylaxis guidelines in trauma: The consequences of noncompliance	Journal of Trauma and Acute Care Surgery	Retrospective review	USA	2012/08	20
90	Point prevalence study of antimicrobial use among hospitals across Botswana; findings and implications	Expert Review of Anti-infective Therapy	Surgery research	Sweden	2019/07	19
91	Hospital-Associated Infections in Small Animal Practice	Veterinary Clinics of North America-Small Animal Practice	Practice guidline	USA	2015/03	19
92	Update on Infection Control Practices in Cancer Hospitals	CA-A Cancer Journal for Clinicaians	Practice guidline	USA	2018/09-11	18
93	Effect of an antibiotic checklist on length of hospital stay and appropriate antibiotic use in adult patients treated with intravenous antibiotics: a stepped wedge cluster randomized trial	Clinical Microbiology and Infection	Stepped wedge cluster randomized trial	Netherlands	2017/07	18
94	Effect of adding a mobile health intervention to a multimodal antimicrobial stewardship programme across three teaching hospitals: an interrupted time series study	Journal of Antimicrobial Chemotherapy	Interrupted time series study	UK	2017/06	18
95	Educational Antimicrobial Stewardship Intervention Ineffective in Changing Surgical Prophylactic Antibiotic Prescribing	Surgical Infections	Before-after study	Australia	2016/04	18
96	Converting Emergency Pilonidal Abscess Into an Elective Procedure	Diseases of the Colon & Rectum	Prospective cohort study	UK	2012/06	18

97	Antibiotic stewardship programmes: legal framework and structure and process indicator in Southwestern French hospitals, 2005-2008	Journal of Hospital Infection	Practice Guideline	France	2011/02	18
98	Impact of pharmacist-led antibiotic stewardship interventions on compliance with surgical antibiotic prophylaxis in obstetric and gynecologic surgeries in Nigeria	Plos One	Prospective study	Nigeria	2019/03	17
99	Exploring the behavioural drivers of veterinary surgeon antibiotic prescribing: a qualitative study of companion animal veterinary surgeons in the UK	BMC Veterinary Research	Research article	UK	2018/11	17
100	Using Bacterial Fluorescence Imaging and Antimicrobial Stewardship to Guide Wound Management Practices: A Case Series	Ostomy Wound Management	Research article	Canada	2018/08	17

Supplementary Table S2. The articles applied AMS and deferred AMR

Rank	Title	Journal	Article type	Study results for AMR	Citations
1	Impact of guidelines and enhanced antibiotic stewardship on reducing broad-spectrum antibiotic usage and its effect on incidence of Clostridium difficile infection	Journal of antimicrobial chemotherapy infection control	Retrospective, quasi-experimental study	Reduced the incidence of Clostridium difficile infection	120
2	Audit and Feedback to Reduce Broad-Spectrum Antibiotic Use among Intensive Care Unit Patients: A Controlled Interrupted Time Series Analysis	Infection control and hospital epidemiology	Prospective, controlled interrupted time series.	ReduceD the incidence of Clostridium difficile infection and overall gram-negative susceptibility to meropenem increased	114
3	Prevalence and Risk Factors for Acquisition of Carbapenem-Resistant Enterobacteriaceae in the Setting of Endemicity	Infection control and hospital epidemiology	Cohort study with a nested case-control study	Control of Carbapenem-Resistant Enterobacteriaceae	112
4	Clostridium difficile disease: Diagnosis, pathogenesis, and treatment update	Surgery	Review	Reduced the incidence of Clostridium difficile infection	53
5	Microbiology and Antibiotic Management of Orbital Cellulitis	Pediatrics	Retrospective chart review	Limited the development of resistant organisms and facilitate transition to an oral agent.	48
6	Infection Reduction Strategies Including Antibiotic Stewardship Protocols in Surgical and Trauma Intensive Care Units Are Associated with Reduced Resistant Gram-Negative Healthcare-Associated Infections	Surgical infections	Intervention study	Decreased the rate of healthcare-acquired infections to specific MDR gram-negative pathogens: Pseudomonas, Acinetobacter and Enterobacteriaceae.	46
7	Differential outcome of an antimicrobial stewardship audit and feedback program in two intensive care units: a controlled interrupted time series study	BMC infectious diseases	Controlled interrupted time series analysis	No changes in incidence of C. difficile infection or resistance patterns of E. coli and P. aeruginosa.	44
8	An antimicrobial stewardship program improves antimicrobial treatment by	Critical care	Retrospective study	Increase in the treatment of sterile-site	42

	culture site and the quality of antimicrobial prescribing in critically ill patients			cultures and a reduction in the treatment of nonsterile-site cultures.	
9	Impact of a multimodal strategy combining a new standard of care and restriction of carbapenems, fluoroquinolones and cephalosporins on antibiotic consumption and resistance of Pseudomonas aeruginosa in a French intensive care unit	International journal of antimicrobial agents	Retrospective comparative study	Rates of resistant P. aeruginosa and of AmpC-hyperproducing Enterobacteriaceae decreased simultaneously.	14
10	Impact of monitoring surgical prophylactic antibiotics and a computerized decision support system on antimicrobial use and antimicrobial resistance	American journal of infection control	Interrupted time series	Trends of the proportions of extended-spectrum β-lactamase–producing Escherichia coli, meropenem-resistant Pseudomonas aeruginosa, and methicillin-resistant Staphylococcus aureus have been reversed or decreased.	14
11	Evaluating the Effectiveness of an Antimicrobial Stewardship Program on Reducing the Incidence Rate of Healthcare-Associated Clostridium difficile Infection: A Non-Randomized, Stepped Wedge, SingleSite, Observational Study	Plos one	Single-site study	Did not seem to reduce the risk of Clostridium difficile infection on the surgery wards	13
12	Impact of antimicrobial stewardship managed by clinical pharmacists on antibiotic use and drug resistance in a Chinese hospital, 2010-2016: a retrospective observational study	BMJ open	Retrospective observational study	1.the resistance rates of E. coli and P. aeruginosa to fluoroquinolones decreased<br>2. the incidence rate of methicillin-resistant Staphylococcus aureus also decreased<br>3.The resistance rates of E. coli and K. pneumoniae to carbapenems increased.	12

13	Reduced Incidence of Carbapenem-Resistant Klebsiella pneumoniae Infections in Cardiac Surgery Patients after Implementation of an Antimicrobial Stewardship Project	Antibiotics-basel	Retrospective observational study	Reduced Incidence of Carbapenem-Resistant Klebsiella pneumoniae Infections in Cardiac Surgery Patients	5
14	Antimicrobial Stewardship Lessons From Mupirocin Use and Resistance in Methicillin-Resitant Staphylococcus Aureus	Open forum infectious diseases	Retrospective study	When consumption was ≤25 DDD/1000 patient-days, Staphylococcus Aureus was decreased in mupirocin resistance.	5
15	Changes in antimicrobial susceptibility of commonly clinically significant isolates before and after the interventions on surgical prophylactic antibiotics (SPAS) in Shanghai	Brazilian journal of Microbiology	Interrupted time series study	The total proportions of MRSA, VRE, IREC, IRKP, IRAB and IRPA in Surgical Department were more than those in ICU and in Internal Department; MRSA has decreased	3
16	Impact of an Institutional Antimicrobial Stewardship Program on Bacteriology of Surgical Site Infections in Cardiac Surgery	Journal of cardiac surgery	Before and after comparative study	The proportion of gram positive, gram negative, fungal, and anaerobic organisms was not significantly different.	3
17	Implementing a combined infection prevention and control with antimicrobial stewardship joint program to prevent caesarean section surgical site infections and antimicrobial resistance: a Tanzanian tertiary hospital experience	Antimicrobial resistance and infection control	Before and after comparative study	A low prevalence of gram-positive isolates and of methicillin-resistant Staphylococus aureus was detected.	0

AMS: antimicrobial stewardship, AMR: antimicrobial resistance, MDR: multi-drug resistance, DDD: defined daily dose; MRSA: methicillin-resistant Staphylococcus aureus, VRE: vancomycin-resistant Enterococcus spp., IREC: imipenem-resistant Escherichia coli, IRKP: imipenem-resistant Klebsiella pneumoniae, IRAB: imipenem-resistant Acinetobacter baumannii; IRPA: imipenem-resistant Pseudomonas aeruginosa

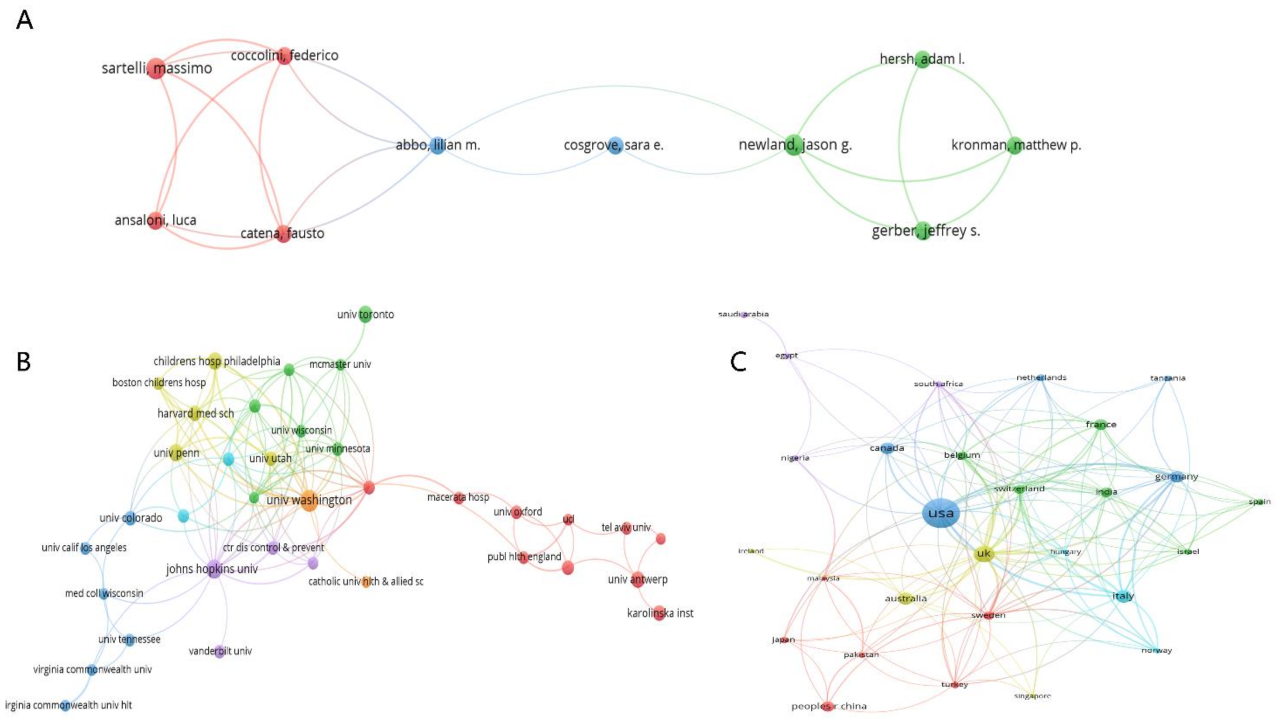

Figure S1 Bibliometric analysis of the co-authorship. (A) The co-authorship map of authors which indicates cooperation of authors in the field of AMS; (B) the co-authorship map of organizations. University of Washington has the most cooperates (19 institutions) and published 17 related papers; (C) the co-authorship map of countries. Six clusters with different colors were shown. The number of collaborators with USA (in bule) is 21 with link strength 56, the number of collaborators with UK (in yellow) is 23 with link strength 84. The size of nodes demonstrates the number of publications and the thickness of the lines indicate the link strength of the countries.

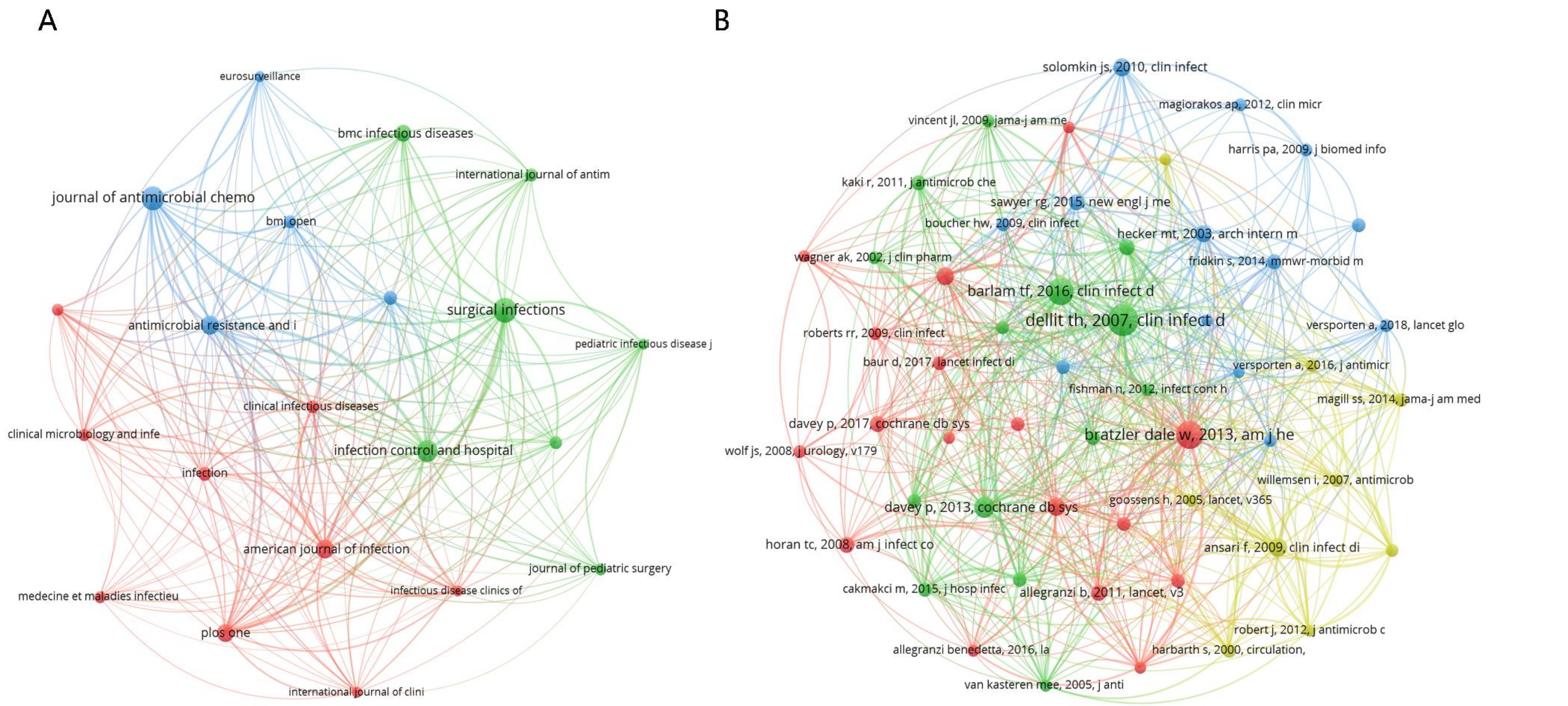

Figure S2 Bibliometric analysis of coupling. (A) bibliographic coupling map of sources; (B) co-citation map of references.

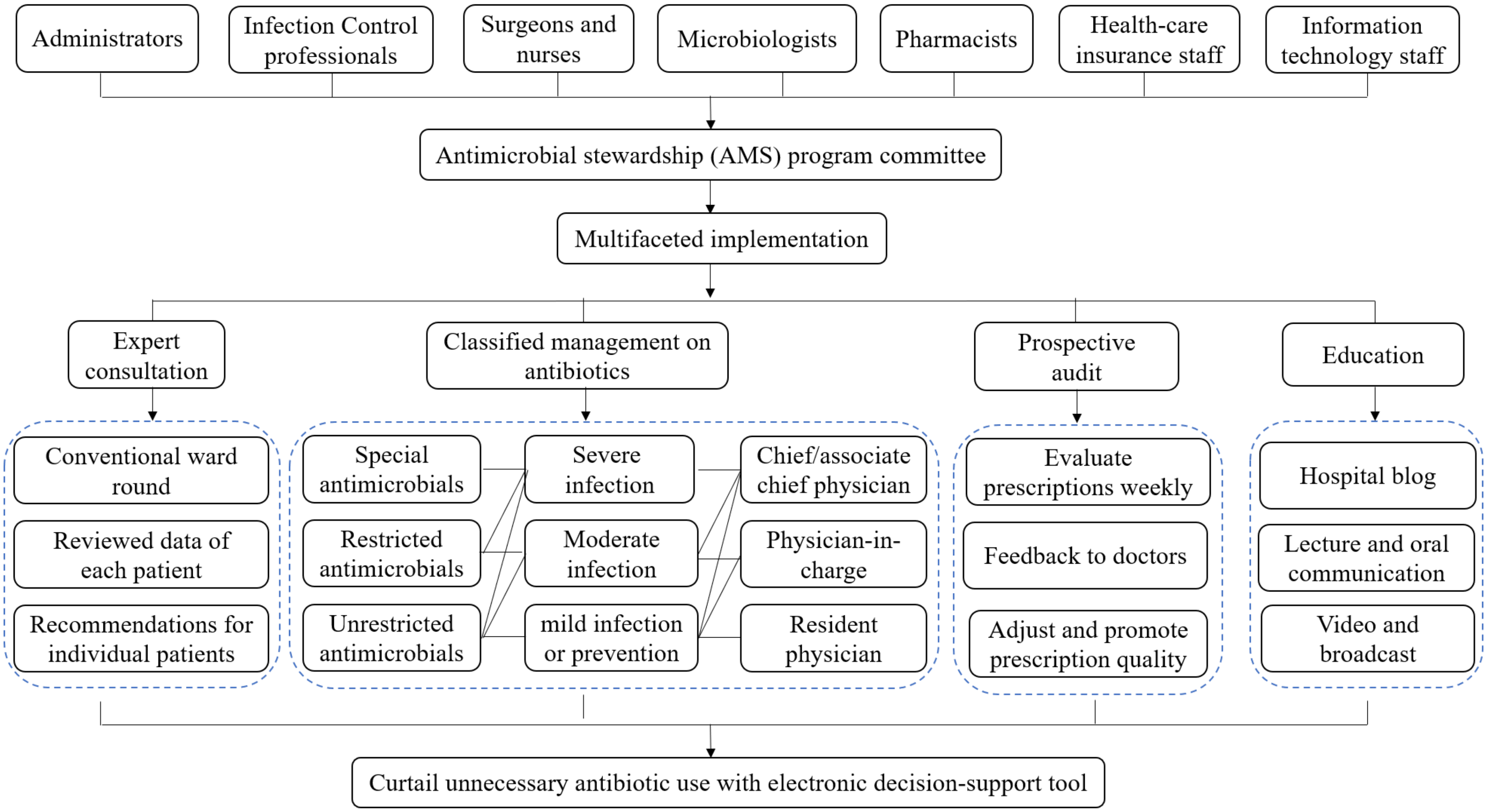

Figure S3 Strategies for implementing antimicrobial stewardship (AMS)
